# Are there any completely sterile organs or tissues in the human body? Is there any sacred place?

**DOI:** 10.1111/1751-7915.14442

**Published:** 2024-03-11

**Authors:** Alfredo Michán‐Doña, Mari C. Vázquez‐Borrego, Carmen Michán

**Affiliations:** ^1^ Departamento de Medicina Hospital Universitario de Jerez Jerez de la Frontera Spain; ^2^ Biomedical Research and Innovation Institute of Cadiz (INiBICA) Cádiz Spain; ^3^ Instituto Maimónides de Investigación Biomédica de Córdoba (IMIBIC) Córdoba Spain; ^4^ Departamento de Bioquímica y Biología Molecular, Campus de Excelencia Internacional Agroalimentario CeiA3 Universidad de Córdoba Córdoba Spain

## Abstract

The human microbiome comprises an ample set of organisms that inhabit and interact within the human body, contributing both positively and negatively to our health. In recent years, several research groups have described the presence of microorganisms in organs or tissues traditionally considered as ‘sterile’ under healthy and pathological conditions. In this sense, microorganisms have been detected in several types of cancer, including those in ‘sterile’ organs. But how can the presence of microorganisms be detected? In most studies, 16S and internal transcribed spacer (ITS) ribosomal DNA (rDNA) sequencing has led to the identification of prokaryotes and fungi. However, a major limitation of this technique is that it cannot distinguish between living and dead organisms. RNA‐based methods have been proposed to overcome this limitation, as the shorter half‐life of the RNA would identify only the transcriptionally active microorganisms, although perhaps not all the viable ones. In this sense, metaproteomic techniques or the search for molecular metabolic signatures could be interesting alternatives for the identification of living microorganisms. In summary, new technological advances are challenging the notion of ‘sterile’ organs in our body. However, to date, evidence for a structured living microbiome in most of these organs is scarce or non‐existent. The implementation of new technological approaches will be necessary to fully understand the importance of the microbiome in these organs, which could pave the way for the development of a wide range of new therapeutic strategies.

## INTRODUCTION

The World Health Organization (WHO) defines the sterility term as ‘the freedom from the presence of viable microorganisms’. Thus, an environment should be considered sterile if there are no viable microorganisms present. But since most microorganisms cannot be successfully cultured using standard techniques, how can we be sure of the absence of viable microorganisms? Perhaps we simply have not been able to detect their growth or to grow them outside their natural environment?

**FIGURE 1:**
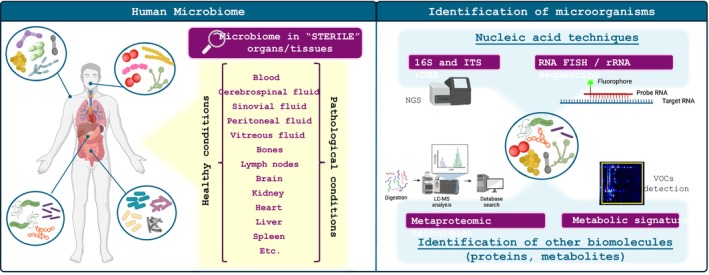
Representative image of the human microbiome and the ‘sterile’ organs or tissues related to the presence of microbiome (right panel) as well as the different techniques described for its identification (left panel). NGS, next‐generation sequencing; ITS, internal transcribed spacer; VOCs, volatile organic compounds.

At birth, humans are colonized with a pleiotropic group of microorganisms that establish a symbiotic association and contribute to essential functions (Lopez et al., 2021). This so‐called human microbiome comprises a huge set of organisms inhabiting and interacting within the human body that contribute to our health, both positively and negatively (Figure 1). This microbiome changes and evolves with us depending on age, gender, environment (nutrition and lifestyle), hormonal changes, inherited genes and underlying diseases (Ogunrinola et al., 2020).

However, there are some organs or tissues in our body that have traditionally been considered sterile, that is blood, cerebrospinal fluid, pleural fluid, peritoneal fluid, pericardial fluid, bone, synovial fluid, lymph nodes, brain, heart, liver, spleen, vitreous fluid, kidney, pancreas, ovary or vascular tissue (Figure 1). This assumption is slowly changing as several papers have been published addressing the presence of multiple microorganisms in some of these human environments, under both physiological and pathological conditions (see references in the following section).

## THE HUMAN MICROBIOME AND HEALTH: EVEN ‘STERILE’ ORGANS OR TISSUES ARE INVOLVED

The human body is populated by myriads of microorganisms throughout its surface and in the cavities connected to the outside. For some years now, the composition, function and diversity of the gut microbiome have been associated with several chronic diseases that also influence distant organs, mucosa and immune function (Vijay & Valdes, 2022). Furthermore, unique microbial signatures have been associated with several diseases, including several types of cancer (see a very nice recent analysis in (Turjeman & Koren, 2022)). Similarly, as there is growing evidence for the microbial presence in organs previously known as sterile sites, we can expect that alterations in these microbiomes should at least functionally influence their host organs.

A few months ago, up to 117 microbial species were identified in the blood of healthy humans, some of which presented evidence of microbial replication, although their presence was rare and sporadic. Furthermore, the blood microbiome did not have a common structure and was not related to the host phenotypes, suggesting that the microbes originate from other body sites but are somehow able to sporadically translocate into the bloodstream (Tan et al., 2023). On the other hand, in a non‐healthy context, infections by Chlamydia strains (among other bacteria) have been associated with several diseases such as coronary atherosclerosis, endocarditis or meningitis. These microbes have been detected in ‘sterile’ organs or tissues as blood (Smolejová et al., 2023), heart (Mavrogeni et al., 2009, 2011) or brain (Piekut et al., 2022) by immunological or PCR techniques. Chlamydia and *Helicobacter pylori* infections have been linked to atheroma disruption in the course of acute infections and to chronic atherosclerotic processes by promoting inflammation (Shah, 2019; Zhang et al., 2019). Nonetheless, there is no definitive evidence that the aforementioned microorganisms are alive and proliferating in these target organs.

One of the current hot topics in clinical science is the relationship between cancer and the microbiome. Carcinogenesis follows three successive steps: initiation, promotion and progression, all of which can be influenced by the microbiota, as microorganisms can: induce mutations, alter the balance between cell proliferation/apoptosis, increase inflammation or contribute to immune evasion (Lopez et al., 2021). Furthermore, microorganisms can also alter the metabolism of anticancer drugs, affecting the chances of beating the disease. The presence of tumours also appears to promote microbial colonization of proximal organs, probably through mucosal destruction (Yang et al., 2023). Note that not all of these effects are negative, and some microbial profiles may be associated with better disease prognosis or directly with tumour inhibition (Yang et al., 2023). The presence of microorganisms has been detected in several types of cancer, including those in ‘sterile’ organs, mostly by immunohistochemistry or sequencing techniques, although these may not be the most suitable techniques for detecting living microbes, as we will discuss later. Nevertheless, a clear proof of how the microbiome, specifically the mycobiome, of a ‘sterile’ organ can influence cancer progression has been reported by Alam et al. (Alam et al., 2022). This work describes how, in mouse pancreatic ductal adenocarcinoma, the intratumoral mycobiome activates a signalling pathway that accelerates pancreatic cancer progression, but also how survival rate can be increased by antifungal treatments (Alam et al., 2022).

## IDENTIFICATION OF MICROORGANISMS USING NUCLEIC ACID SEQUENCING TECHNIQUES

In most studies, the presence of microorganisms has been investigated by sequencing their 16S or their internal transcribed spacer (ITS) ribosomal DNA (rDNA) (Figure 1), leading to the identification of prokaryotes and fungi, respectively (Regueira‐Iglesias et al., 2023). The rDNA sequencing has several advantages, as it is easy and cheap to perform, and bioinformatic analysis is also well established. New next‐generation sequencing techniques, including those specifically designed to improve the analysis of metagenomes such as Mobimicrobe and 2b‐RAD‐M, although generally more expensive, can improve the depth of the analysis, easily reaching the species level (Yi et al., 2024). However, a major limitation of these techniques is that it cannot distinguish between living and dead organisms and that in complex samples, results can be biased by poor DNA quality or external DNA contamination (Wang et al., 2023). RNA‐based methods, such as RNA fluorescence in situ hybridization or rRNA sequencing, have been proposed to overcome the first limitation, as the shorter half‐life of the RNA would only identify the transcriptionally active microorganisms, although perhaps not all the viable ones. Nevertheless, rRNAs are the most stable RNAs and can remain in the environment long after cell death. Furthermore, many biochemical factors can influence the stability of 16S RNA‐seq readouts, so their abundance may not reflect the real number of bacterial counts. There are other highly conserved bacterial RNAs with shorter half‐lives that have been proposed as markers for living microorganisms, but their sequence correspondence with the different species is not as well established, making microbial identification difficult (Wang et al., 2023). Finally, a bottleneck that is not usually considered is that many of the dormant microorganisms have a really hard to break down cell wall, which can make nucleic acid extraction difficult. For this reason, many microbial studies from highly complex environments show conflicting results, simply due to differences in the efficiency of their nucleic acid isolation procedures.

## IDENTIFICATION OF MICROORGANISMS BY OTHER TECHNIQUES

What are the alternatives? Either we move on to the identification of other biomolecules, such as proteins or metabolites (Figure 1), or we move on to other more specific but less global techniques. Metaproteomic techniques could be a really good alternative to identify living microorganisms, because proteins are specific and have a limited half‐life, although they will only give us a qualitative picture of living taxa, as their concentration varies between different taxonomic units as already discussed in the ‘Crystal Ball’ section of Environmental Microbiology (Armengaud, 2023). Protein isolation techniques are not as well standardized as those for nucleic acids, so isolating proteins from complex matrices can be a real challenge. On top of this, there are no good global microbial protein databases that can be used to identify the isolated peptide spectra. In our group we have used separate databases to investigate the microbiome of Pseudomyxoma peritonei, a rare type of peritoneal mucinous neoplasm, but the results obtained may be redundant and not able to clearly separate human from microbial proteins (Fuentes‐Almagro, Pezzopane, Alhama, Michán, Arjona‐Sánchez, Vázquez‐Borrego & Romero‐Ruiz, unpublished results). In addition, the diversity of protein sequences does not usually allow us to distinguish between lower taxonomic levels, for example species. Other possibilities that we are also exploring include the search for molecular metabolic signatures, or the use of microscopy combined with specific staining techniques (Salatti‐Dorado, Bura, Alhama, Michán, Arjona‐Sánchez, Vázquez‐Borrego & Romero‐Ruiz, unpublished results), although both approaches are not as global and require prior knowledge of what we are looking for. Last but not least, we should mention the use of MALDI Imaging Spectrometry (MSI) in combination with other microscopic techniques that could provide a molecular identification of non‐human cells in a human tissue/organ.

## WHAT NEXT?

New technological advances are challenging the notion of ‘sterile’ organs in our bodies. However, to date, evidence for a structured living microbiome in most of these organs is scarce or non‐existent. To fully understand the importance of the microbiome of these organs and its influence on human health/disease, we need to be sure of its real composition, its viability and its functionality. Much work remains to be done, which will probably require the implementation of more than one technological approach, to demonstrate the existence of these microbiomes and their relationship with the different pathological conditions affecting their host organs. However, the benefits could be enormous, as understanding the structure of the human microbiome in the different organs or tissues and its relationship to their functionality could open the door to a wide range of new therapeutic strategies that could certainly improve our quality of life.

All comments in this manuscript refer to cellular microorganisms, as the human virome is much larger, variable, complex and unknown.

## AUTHOR CONTRIBUTIONS

**Alfredo Michán‐Doña:** Conceptualization; visualization; writing – original draft. **Mari C. Vázquez‐Borrego:** Conceptualization; project administration; visualization; writing – original draft; writing – review and editing. **Carmen Michán:** Conceptualization; project administration; visualization; writing – original draft; writing – review and editing.

## CONFLICT OF INTEREST STATEMENT

The authors have no conflict of interest to declare.
